# MARGRA Lamb Eating Quality and Human Health-Promoting Omega-3 Long-Chain Polyunsaturated Fatty Acid Profiles of Tattykeel Australian White Sheep: Linebreeding and Gender Effects

**DOI:** 10.3390/antiox9111118

**Published:** 2020-11-12

**Authors:** Shedrach Benjamin Pewan, John Roger Otto, Robert Tumwesigye Kinobe, Oyelola Abdulwasiu Adegboye, Aduli Enoch Othniel Malau-Aduli

**Affiliations:** 1 Animal Genetics and Nutrition, Veterinary Sciences Discipline, College of Public Health, Medical and Veterinary Sciences, Division of Tropical Health and Medicine, James Cook University, Townsville, QLD 4811, Australia; shedrach.pewan@my.jcu.edu.au (S.B.P.); john.otto@jcu.edu.au (J.R.O.); robert.kinobe@jcu.edu.au (R.T.K.); 2 National Veterinary Research Institute, Private Mail Bag 01 Vom, Plateau State, Nigeria; 3 Australian Institute of Tropical Health and Medicine, College of Public Health, Medical and Veterinary Sciences, Division of Tropical Health and Medicine, James Cook University, Townsville, QLD 4811, Australia; oyelola.adegboye@jcu.edu.au

**Keywords:** antioxidants, Tattykeel Australian White, MARGRA lamb, meat quality, *longissimus dorsi* muscle, omega-3 LC-PUFA, fat melting point, intramuscular fat, inbreeding coefficient, gender

## Abstract

Health-conscious consumers increasingly demand healthier, tastier, and more nutritious meat, hence the continuous need to meet market specifications and demand for high-quality lamb. We evaluated the *longissimus dorsi* muscle of 147 Tattykeel Australian White (TAW) sheep fed on antioxidant-rich ryegrass pastures exclusive to MAGRA lamb brand for meat eating quality parameters of intramuscular fat (IMF) content, fat melting point (FMP) and omega-3 long-chain polyunsaturated fatty acids (n-3 LC-PUFA). The aim was to assess the impact of linebreeding and gender on pasture-fed lamb eating quality and to test the hypothesis that *variation in healthy lamb eating quality is a function of lamb gender and not its antioxidant status or inbreeding coefficient* (IC). After solid-phase extraction and purification, phenolics and antioxidant enzyme activities were analysed by high-performance liquid chromatography and mass spectrometry. IMF and fatty acid composition were determined using solvent extraction and gas chromatography, respectively. IC was classified into low (0–5%), medium (6–10%) and high (>10%) and ranged from 0–15.6%. FMP and IMF ranged from 28 to 39 °C and 3.4% to 8.2%, with overall means of 34.6 ± 2.3 °C and 4.4 ± 0.2%, respectively, and n-3 LC-PUFA ranged from “source” to “good source” levels of 33–69 mg/100 g. Ewes had significantly (*P* ˂ 0.0001) higher IMF, C22:5n-3 (DPA), C22:6n-3 (DHA), C18:3n-6, C20:3, C22:4n-6, C22:5n-6, total monounsaturated (MUFA), PUFA and Σn-3 fatty acids and lower total saturated fatty acids (SFA) and FMP, than rams. As IC increased, there were no differences in FMP and IMF. Folin–Ciocalteu total phenolics, ferric reducing antioxidant power and antioxidant activities of glutathione peroxidase, catalase and superoxide dismutase enzymes did not differ by either gender or IC. This study provides evidence that IC is inconsequential in affecting antioxidant status, IMF, FMP and n-3 LC-PUFA in linebred and pasture-fed TAW sheep because the observed variation in individual fatty acids was mainly driven by gender differences between ewes and rams, hence the need to accept the tested hypothesis. This finding reinforces the consistent healthy eating quality of MARGRA lamb brand from TAW sheep regardless of its linebred origin.

## 1. Introduction

The Food and Agriculture Organisation (FAO) of the United Nations defines meat quality as the constitutional standard of lean-to-fat ratio and palatability indices that include visual appearance, aroma, drip loss, colour, texture, pH, intramuscular fat content, fatty acid and fat melting point profiles, tenderness, flavour and juiciness [1]. Meat and Livestock Australia (MLA) describes the entire processes of feeding culminating in the finishing of animals including their genetic constitution, husbandry practices and handling, as all affect the overall quality of meat [2]. Fat melting point (FMP), intramuscular fat (IMF) content (marbling) and fatty acid (FA) profile all influence eating quality and, ultimately, consumer preferences for consistent, safe, nutritious and tasty lamb with a healthy FA composition [3]. Globally, meat is regarded as one of the main sources of animal protein [4,5] and lamb is known to be highly nutritious and digestible [6], fortified with essential amino acids, iron, zinc, selenium, fatty acids and vitamins A, B6 and B12 [5]. Lamb also has relatively low lipid and saturated fat contents compared to meat from other ruminants [7], and its marbling, tenderness, juiciness, aroma and colour attributes have been known to influence consumer liking [8], carcass [9], meat assignment into quality grades [10], consumer food choices [11] and nutritional value [12]. It is therefore very important that sheepmeat producers guarantee the consistency of their lamb products in order to meet consumer preferences and adapt to the dynamics of purchasing decisions based on meat eating quality.

FMP dictates fat firmness. Soft fat has a low melting point and vice versa [13]. From a nutritional perspective, fats with low melting points consist of high levels of unsaturated fatty acids, and, conversely, fats with high melting points have comparably higher saturated fatty acids [14,15]. IMF content or marbling is a main determinant of meat eating quality in most carcass grading systems [16]. As the IMF increases, so does the eating quality [17] because it influences meat palatability and contributes significantly to juiciness, flavour and tenderness [18,19]. Consumers therefore prefer meat with low FMP, moderate IMF and fatty acid composition with proportionately more of the health-promoting omega-3 long-chain polyunsaturated fatty acids (n-3 LC-PUFA). Given that humans and other vertebrates lack the capacity to synthesize n-3 LC-PUFA because they lack the enzyme Δ^15^ desaturase, they must obtain these from dietary intake sources in order to meet their daily requirement of 500 mg of n-3 LC-PUFA [20]. Lamb producers can tap into the omega-3 functional meat market niche by matching their sheep breeding and production system to meet this health-conscious consumer preference. 

Ryegrass is a popular grass species in pasture-based grazing production systems in Australia and New Zealand. Ryegrass contains many phenolic compounds such as gallic and salicylic acids (phenolic acids), tannins, coumarins, flavonoids, α-tocopherol, lignans, xanthones and anthocyanidins [21,22]. These phenolic compounds in ryegrass serve as natural antioxidants, anti-inflammatory and anti-septic agents [23] that enhance meat oxidative stability and quality attributes such as nutritive value, flavour and colour. Luciano et al. [24,25] reported that antioxidants in dietary tannins from fresh herbage improved colour stability in Comisana lambs by halting myoglobin oxidation in the muscle and reducing meat colour deterioration. In lambs grazing ryegrass, phenolics and antioxidant enzyme activities have been demonstrated to impact oxidative stability in the *Longissimus thoracis et lumborum* muscle [26], liver and plasma [27]. Research investigations of perceived sheepmeat eating quality sensory scores [28] and demographic influences [29] on Australian, American and Chinese consumers demonstrated a consistent consumer response to production factors of muscle type, sire, age, and sex. Evidence in the published literature [30] indicates that meat eating quality and fatty acid (FA) composition of lipids in tandem with variable fat deposition at the attainment of maturity, vary in the muscles of sheep due to differences in breed [31,32,33,34], physiological status, breeding systems [35], grass-fed versus concentrate feeding [36,37], and sex [38,39]. 

Linebreeding is a sheep breeding practice of mating closely related animals that can be traced back to one common ancestor with highly desirable attributes. The Tattykeel Australian White (TAW) sheep are renowned for producing the remarkably unique high-eating-quality MARGRA lamb brand, and were developed from more than a decade of rigorous selection, culling and linebreeding of Texel, Van Rooy, Dorper and Poll Dorset with an extensive utilisation of natural mating, artificial insemination and embryo transfer. Linebreeding increases the frequency of desirable alleles, selection intensity and homozygosity, hence a tight culling regime and close monitoring of the inbreeding coefficient are key breeding management practices that ensure uniformity and consistency in TAW lamb eating quality. A comprehensive review of omega-3 long-chain polyunsaturated fatty acids (n-3 LC-PUFA) metabolism and meat eating quality in TAW lambs [30] previously identified knowledge gaps in using *Longissimus dorsi* muscle biopsy sampling of ram and ewe lambs to directly determine the impact of linebreeding and gender on n-3 LC-PUFA, IMF and FMP contents while the animals are young and alive for early selection and breeding purposes. It also recommended the need for further research to better understand the genetic and nutritional interactions between dietary n-3 LC-PUFA oil supplements versus pasture grazing, finishing performance, carcass traits and the unique eating quality of TAW lambs to afford industry players the opportunity to consistently meet consumer preferences as well as key demand and supply determinants of profitability. This paper aims to fill some of these knowledge gaps by assessing the impact of linebreeding and gender on pasture-fed lamb eating quality consistency in antioxidant status, IMF, FMP, n-3 LC-PUFA and to test the hypothesis that variation in healthy lamb eating quality will be a function of lamb gender and not its antioxidant status or inbreeding coefficient (IC) as an index of linebreeding. 

## 2. Materials and Methods 

### 2.1. Animal Ethics

The use of animals and all procedures performed in this study were approved by the James Cook University Animal Ethics Committee (Permit No. A0015657) in compliance with the Australian Code for Care and Use of Animals for Scientific Purposes (Eighth edition, 2013).

### 2.2. Animals and Experimental Design

**Figure 1:**
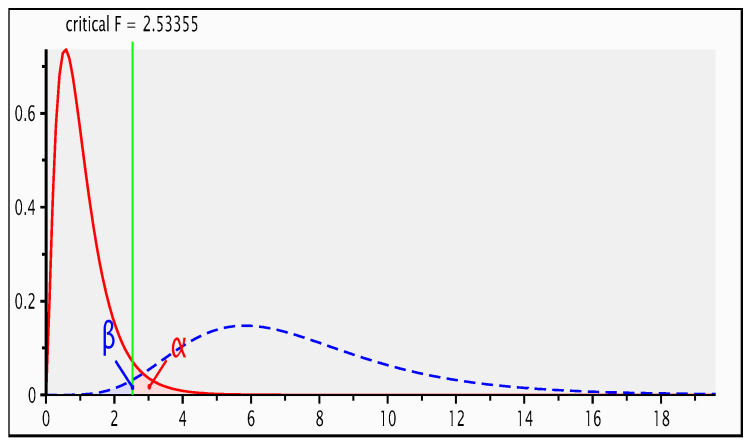
G-Power analysis for statistical power, effect and sample size.

The animals used in this study comprised a cohort of 100 ewe and 47 ram lambs at the Tattykeel Australian White stud farm in Black Springs, Oberon, New South Wales, Australia, grazing the same ryegrass pastures in separate paddocks. They were all 10-month-old lambs, with an average liveweight of 36.8 ± 0.3 kg (range of 36–38 kg for rams), 37.4 ± 0.4 kg (range of 37–38 kg for ewes), and an overall mean body condition score of 2.5 ± 0.01. Carcass performance and meat quality characteristics of TAW had been published [30]. An a priori power analysis was conducted using G-Power to justify an appropriate sample and effect size. As depicted in Figure 1, to achieve a statistical power of 95% with a critical F-value of 2.5, a minimum total sample size of 146 lambs was sufficient for a large effect size, and a two-sided significance level of 0.05. Therefore, the cohort of 100 ewe and 47 ram lambs at the Tattykeel Australian White stud farm in Black Springs, Oberon, New South Wales, Australia grazing the same ryegrass pastures in separate paddocks used in this study, was a sufficient and statistically robust experimental design. Total digestible nutrients [40] and metabolisable energy [41] were computed from the nutritive composition of the ryegrass (Table 1) analysed by the Association of Official Analytical Chemists (AOAC) wet chemistry procedure. 

### 2.3. Muscle Biopsy Sampling Procedure

**Figure 2:**
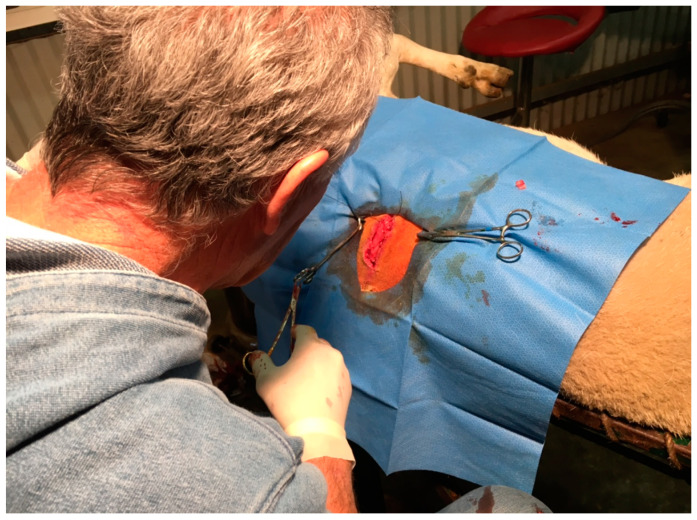
Muscle biopsy sampling technique in Tattykeel Australian White sheep.

*Longissimus dorsi* muscle biopsy samples were taken from the 12th–13th rib interface following the procedure described by Malau-Aduli et al. [42] and are shown in Figure 2. Briefly, the animal was directed into a weighing chute with collapsible sides and some head restraint. The *Longissimus dorsi* muscle area on the back of the animal between the 12th and 13th ribs was shaved with a small electric clipper and cleaned with 90% ethanol and chlorhexidine. About 15 mL of a local anaesthetic agent, lignocaine was administered intramuscularly. Five minutes following the administration of the anaesthetic, a 5–7 cm incision was made with a scalpel blade and about 5 g of the underlying fat and *Longissimus dorsi* muscle was sampled. The wound was closed via 3–4 interrupted sutures using surgilon thread. An anti-bacterial aerosol, Cetrigen, was applied to the sutured area on the skin to promote wound healing, prevent flies and the animal was released back to the paddock. No post-operative complications were reported as healing was rapid. The sutures were removed after 10–14 days. The muscle biopsy sample was immediately placed in a plastic bag on dry ice, flushed with nitrogen gas and transferred into a mobile refrigerator. Samples were transported frozen and stored at −20 °C pending further analysis in the laboratory. The muscle biopsies were analysed for IMF content, FMP and FA composition.

### 2.4. Determination of Intramuscular Fat

The procedures of Holman et al. [43] and Flakemore et al. [44] were utilised for IMF determination. Briefly, the muscle sample was homogenised and 1g transferred to a labelled 50 mL plastic tube containing 20 mL of chloroform: methanol (2:1) solvent and shaken vigorously for 5 min. A filter paper was used to collect the filtrate in another labelled 50 mL tube. Approximately 5 mL of 10% KCl was added to the filtrate to precipitate and separate the inorganic and lipid fractions into two distinct layers. The upper inorganic layer was removed and discarded, while the lower lipid layer was transferred into a clean, dry, pre-weighed and labelled ceramic crucible and evaporated in a laminar fume hood over a heating block. The crucible was cooled and further dried in a desiccator for 10–20 min before it was re-weighed. Samples were analysed in duplicates to allow for replication and reproducibility. Intramuscular fat percentage was calculated as:

[(Final crucible weight) − (Initial crucible weight)/(Initial sample weight)] × 100.

### 2.5. Determination of Fat Melting Point

**Figure 3:**
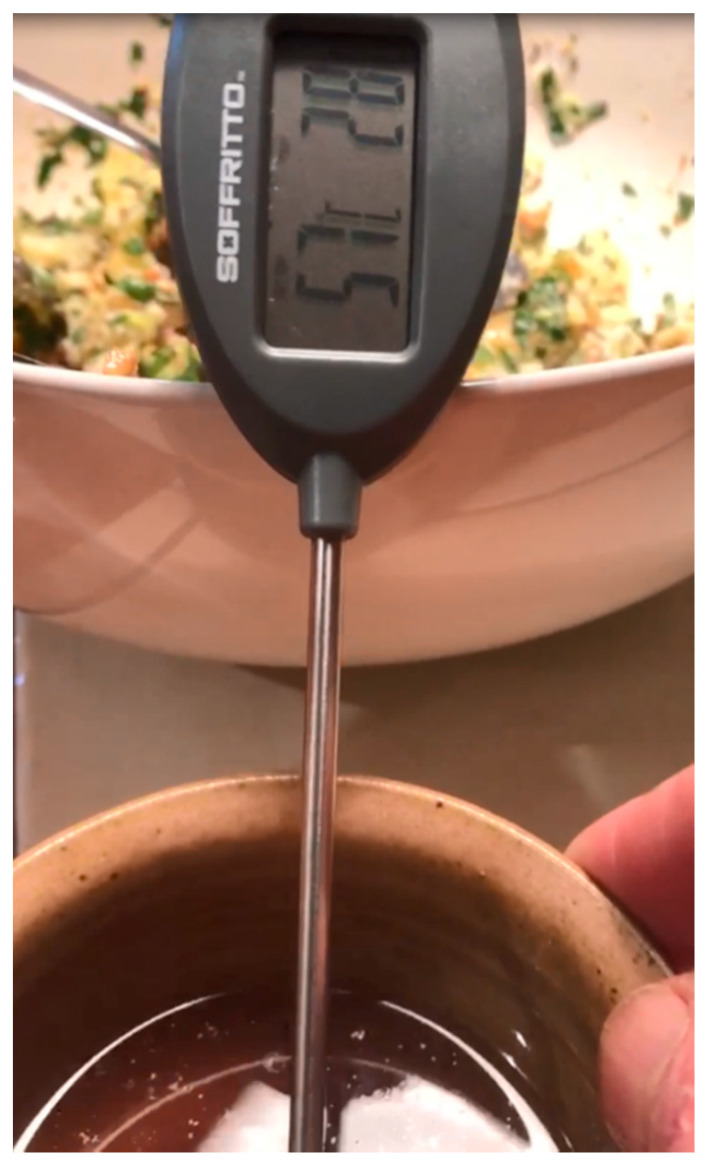
Tattykeel Australian White intramuscular fat (liquid at room temperature) indicating a low fat melting point (FMP).

The procedures of Holman et al. [43] and Flakemore et al. [44] were utilised for FMP determination. Briefly, the crucible containing the extracted IMF was placed in an oven at 100 °C for about 1–2 min to melt the fat. Using air suction, the melted fat was sucked into a thin capillary tube and placed in a refrigerator for about 10 min for the fat to solidify. The fat level in the capillary tube was marked with an indelible pen. The capillary tube was attached to a thermometer and vertically suspended in a beaker containing 80 mL of cold water, gradually heated over a heating block and closely observed until the fat melted and “slipped” (rose above the mark) within the capillary tube. The temperature at which this slip occurred was recorded as the fat melting point. Samples were analysed in duplicates to allow for replication and reproducibility. TAW lamb has a very low fat melting point and can be liquid at room temperature of 25–28 °C as shown in Figure 3.

### 2.6. Determination of Fatty Acid Composition

Fatty acid composition including n-3 LC-PUFA analysis of *Longissimus dorsi* muscle biopsy samples was analysed by means of gas chromatography–mass spectrophotometry procedure described by Malau-Aduli et al. [45]. Briefly, total lipids in 1 g of un-homogenised muscle tissue samples were extracted overnight using a modified Bligh and Dyer [46] method. The first step was a single-phase overnight extraction using CHCl_3_:MeOH:H_2_O (1:2:0.8 *v*/*v*). The second step involved phase separation with the addition of CHCl_3_:saline Milli-Q H_2_O (1:1 *v*/*v*) followed by rotary evaporation of the lower chloroform phase at 40 °C to obtain total lipids. The extracted total lipids were separated into lipid classes by thin layer chromatography (TLC) using 100 mL of the lipid extract reconstituted in hexane [42]. The extract was spotted onto silica gel G plates (200 × 200 × 0.25 mm^3^) with a micropipette. The TLC plate was developed in an acetone/petroleum ether (1:3 vol/vol) solvent system in a tank containing a few crystals of butylated hydroxytoluene (BHT) to prevent oxidation. Triacylglycerols, cholesterol and free fatty acids migrated, while phospholipids remained at the origin of the plate. The areas corresponding to the phospholipids were scraped off the plate and each lipid class transferred to clean screw-capped test tubes for transmethylation and eventual computation of the lipid conversion factor (LCF) of 0.912 on the basis of g fatty acids/g total lipids (0.083 phospholipids, 0.829 triacylglycerols and 0% cholesterol because cholesterol does not contain any fatty acids). An aliquot from each total lipid extract was used for transmethylation with MeOH:CHCl_3_:HCl (10:1:1 *v*/*v*) for 2 h at 80 °C. Fatty acid methyl esters (FAME) were extracted three times using hexane:CHCl_3_ (4:1 *v*/*v*). A known concentration of an internal standard (19:0) was added in a 1500 μL vial containing the extracted FAME. The FAME were analysed on a 7890B gas chromatograph (Agilent Technologies, Palo Alto, CA, USA) equipped with an Equity^TM^-1 fused 15 m silica capillary column with 0.1 mm internal diameter and 0.1 μm film thickness (Supelco, Bellefonte, PA, USA), a flame ionisation detector, a split/splitless injector and an Agilent Technologies 7683 B Series autosampler. The gas chromatograph conditions were: splitless mode injection; carrier gas He; initial oven temperature 120 °C and then increased to 270 °C at flow rates of 10 °C/min and to 310 °C at 5 °C/min. The Agilent Technologies ChemStation software (Palo Alto, CA, USA) was used to quantify fatty acid peaks. The fatty acid identities were confirmed by gas chromatograph–mass spectrometric (GC/MS) analysis using a Finnigan Thermoquest GCQ^TM^ GC/MS fitted with an on-column injector and Thermoquest Xcalibur software (Austin, TX, USA). The gas chromatograph (GC) was equipped with an HP-5 cross-linked methyl silicone-fused silica capillary column (50 m × 0.32 mm internal diameter) which is of similar polarity to the column described above. The carrier gas was helium (head pressure 30 kPa) and GC conditions had been previously described by Miller et al. [47]. Fatty acid percentages were computed as follows: FA% = [(individual fatty acid area) ∗ (100)]/(sum total area of fatty acids). Fatty acid contents were calculated as follows: FA mg/100 g = (Total lipid) ∗ (LCF [0.912]) ∗ ([%FA]/100) ∗ 1000, where 0.912 was the derived lipid conversion factor similar to the one cited by Clayton [48].

### 2.7. Extraction and Purification of Phenolic Compounds

Solid-phase extraction, purification and analysis of phenolics in the ryegrass utilised the procedure described in detail by López-Andrés et al. [27]. Briefly, 2.5 g of the ryegrass was chopped to pass through a 1 mm sieve and homogenised at 4000 r.p.m. in 15 mL of acetone/water 70/30 *v/v* for 1 min and sonicated for 6 min in a water bath. Homogenates were centrifuged at 4 °C for 15 min at 3000× *g* and the supernatants filtered with Whatman filter papers. About 10 mL of the filtered supernatant was acidified with 0.5 M H_2_SO_4_ and loaded onto reversed phase cartridges (C18 Sep-Pak Vac WAT043395, WATERS, Milan, Italy) preconditioned with methanol and distilled water to disrupt polyphenol-binding protein. The extracted phenolics were eluted with 2 mL methanol and stored in a −30 °C freezer until ready for Folin–Ciocalteu (FCTP) and ferric reducing antioxidant power (FRAP) assays using a double-beam spectrophotometer (model UV-1601, Shimadzu Corporation, Milan, Italy) to measure the absorbance of the samples at 725 nm and 593 nm, respectively. Details of both assay procedures have been described [27] and will not be repeated herein.

### 2.8. Antioxidant Enzyme Activities

Antioxidant activities of glutathione peroxidase, catalase and superoxide dismutase enzymes in the muscle were assayed as described by Petron et al. [26]. Briefly, about 5 g of the *Longissimus dorsi* muscle was homogenised in 25 mL of 0.005 M phosphate buffer (pH 7.0) and centrifuged at 4 °C for 20 min at 7000 g. The supernatant fraction was filtered through glass wool and used to determine glutathione peroxidase, catalase and superoxide dismutase enzyme activities. By measuring the inhibition of pyrogallol autoxidation, total superoxide dismutase (SOD) activity (Cu–Zn SOD + Mn SOD) was determined where one unit was taken as the activity that inhibits the reaction by 50% [1]. To determine glutathione peroxidase enzyme activity, the oxidation of NADPH at 22 °C was used. The assay medium (3 mL) consisted of 1 mM reduced glutathione, 0.15 mM NADPH, 0.15 mM H_2_O_2_, 40 mM potassium phosphate buffer (pH 7.0), 0.5 mM EDTA, 1 mM NaN_3_, 1.5 units of glutathione reductase, and 300 μL of the muscle extract. Absorbance at 340 nm was recorded over 3 min. An extinction coefficient of 6300 M^−1^ cm^−1^ was used for calculation of NADPH concentration. One unit of glutathione peroxidase enzyme activity was defined as the amount of extract required to oxidize 1 μmol of NADPH per min at 22 °C [1]. Catalase enzyme activity was performed as described by Petron et al. [1]. About 2 mL of the *Longissimus dorsi* muscle supernatant (2 mL) was reacted at room temperature (∼22 °C) with 1 mL of 30 mM H_2_O_2_ in 0.05 M phosphate buffer (pH 7.0), and the reaction (H_2_O_2_ decomposition) was monitored by measuring the absorbance at 240 nm during the initial 30 s. An extinction coefficient of 0.040 cm^2^ μmol^−1^ was used for calculation of H_2_O_2_ splitting. One unit (U) of catalase activity was defined as the amount of extract needed to decompose 1 μmol of H_2_O_2_ per min [26].

### 2.9. Statistical Analysis

IC as an index of linebreeding, estimates the probability that two alleles in an individual lamb will be homozygous (HH or hh) rather than heterozygous (Hh) because the parents are related and have one common ancestor. In other words, IC measures the extent to which two genes at any locus in an individual lamb are identical by descent from the common ancestor(s) of the two parents. IC was computed as:

F_X_ = Σ[(½)^n+1^ (1 + F_A_)] (1)

where F_x_ = IC of lamb X, Σ = summation, n = number of common ancestors connecting the parents of lamb X and F_A_ is the IC of the common ancestor A.

Fatty acids, IMF, FMP, antioxidants and enzyme activities were analysed as dependent variables using multivariate analysis of variance (MANOVA) after fitting the fixed effects of gender and IC in General Linear Model procedures (PROC GLM) using Statistical Analysis System software (SAS) version 9.4 (SAS Institute, Cary, NC, USA) [49]. First-order interactions between gender and IC were initially tested but later dropped from the final model due to non-significance. The initial full statistical model used for the analysis was:

Y = μ + G_i_ + B_j_ + (GB)_ij_ + e_ijk_ (2)

where Y = dependent variable (FMP, IMF, FA, antioxidants and enzyme activities), μ = overall mean, G_i_ = Gender, B_j_ = Inbreeding Coefficient, (GB)_ij_ = first-order interaction between gender and inbreeding coefficient, and e_ijk_ = residual error. Level of significance threshold was set at *p* < 0.05 and differences between least square means were established using Tukey’s pairwise comparison test.

## 3. Results

### 3.1. Nutrient Composition of the Grazed Ryegrass Pasture, Muscle Phenolics and Antioxidant Enzyme Activities

The ewe and ram lambs utilised in this study grazed high-quality ryegrass whose nutrient composition and antioxidant status is presented in Table 1 and fatty acid profile in Table 2. The low dry matter is indicative of fresh pasture with high moisture content, while the high phenolic antioxidants, crude protein, low neutral detergent and high metabolisable energy are all indicative of high palatability, digestibility and total digestible nutrients from the ryegrass pastures that are typical during spring. There were no significant differences due to gender (Table 3) and inbreeding coefficient (Table 4) in total phenolics and antioxidant enzyme activities of glutathione peroxidase, catalase and superoxide dismutase in the *Longissimus dorsi* muscle of these ryegrass pasture-fed lambs.

### 3.2. Intramuscular Fat Content (IMF)

**Figure 4:**
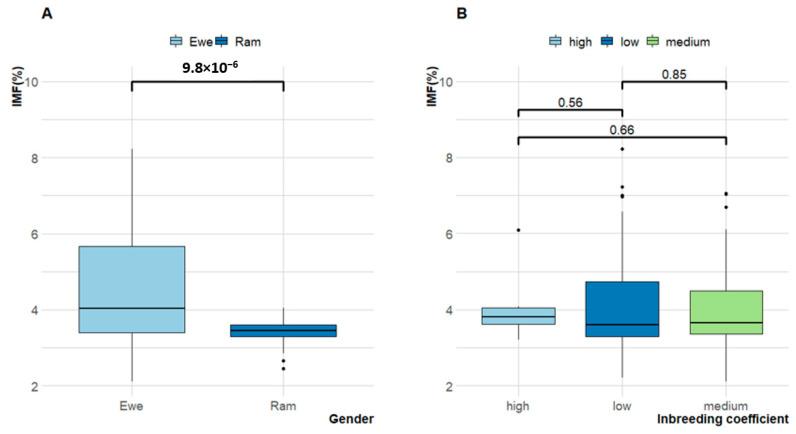
Variation in intramuscular fat (IMF) percentage of Tattykeel Australian White (TAW): (**A**) gender; (**B**) Inbreeding Coefficient (IC).

IMF ranged from 3.4% to 8.2%, but ewe lambs had significantly higher IMF (4.4 ± 1.4%) than ram lambs (3.4 ± 0.3%) as shown in Figure 4A. Irrespective of gender, the overall IMF was 4.1 ± 1.3% (Table 3). As shown in Table 4, IC as an index of linebreeding was classified into low (0–5%), medium (6–10%) and high (>10%) and ranged from 0% to 15.6%. As IC increased, there were no differences in IMF (Table 4 and Figure 4B).

### 3.3. Fat Melting Point (FMP)

**Figure 5:**
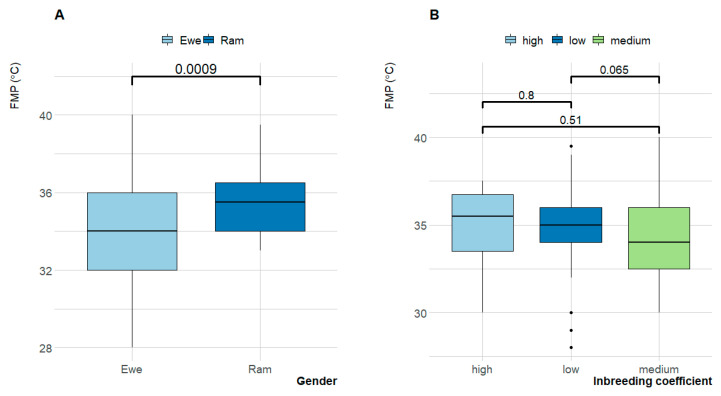
Variation in fat melting point (FMP) percentage of Tattykeel Australian White (TAW): (**A**) gender; (**B**) Inbreeding Coefficient (IC).

FMP ranged from 28 to 39 °C, but ewe lambs had significantly lower FMP (34.26 ± 2.43 °C) than ram lambs (35.5 ± 1.5 °C) as shown in Figure 5. Irrespective of gender, the overall FMP was 34.6 ± 2.3 °C (Table 3). Similar to IMF, IC was not significantly associated with FMP (Table 4 and Figure 4B).

### 3.4. Faty Acid Composition

The fatty acid composition in ewe and ram lambs in mg /100 g tissue is shown in Table 3. It shows that ewe lambs had significantly (*P* ˂ 0.0001) higher C22:5n-3 (DPA), C22:6n-3 (DHA), C18:3n-6, C20:3, C22:4n-6, C22:5n-6, MUFA, PUFA and Σn-3 and lower SFA fatty acids than ram lambs. Although n-3 LC-PUFA ranged from “source” to “good source” levels of 33–69 mg /100 g in individual lambs, overall, there were no gender differences in the health-promoting EPA, DPA, EPA+DHA and EPA+DHA+DPA (Table 3). As IC increased, there were no differences in C20:5n-3 (EPA), DHA, DPA, EPA+DHA, EPA+DHA+DPA and ∑n-6/ ∑n-3 ratio, while increases in C18:3n-3 (ALA), MUFA, PUFA, C18:1, C18:2n-6, C18:3N-6, ∑n-3 PUFA and ∑n-6 PUFA were observed as IC decreased from high to low (Table 4).

## 4. Discussion

Consumer preferences, behaviours, perceptions and satisfaction with the eating quality of meat products are intricately linked to flavour, odour, colour, aroma, taste and juiciness [50,51]. Previous studies [52,53,54,55,56,57] identified diet-related “pastoral flavour” in lamb, also described as “milky”, “barnyard”, “sheepy” or “faecal” flavour, to negatively impact consumer liking. It is thought that this unpleasant “pastoral flavour” originates from skatole (3-methylindole) and indole derivatives from the degradation of tryptophan, 4-methylphenol and other branched chain fatty acids in the rumen [58]. Lamb has also been reported to have a distinct age-related “mutton flavour” and aroma associated with the three branched chain fatty acids 4-methylnonanoic, 4-methyloctanoic and 4-ethyloctanoic acids [59]. Other previous studies had demonstrated that the nutritional background of pasture-fed ruminants confers a higher muscle α-tocopherol antioxidant status compared to those on concentrate based diets [60,61,62,63,64]. Several internal and external factors influence the quantity and quality of lipids in animal products due to genetics-nutrition interactions in the expression of genes controlling fat metabolism [65] and these include the key attributes of fat melting point, intramuscular fat and fatty acid composition. The Folin–Ciocalteu total phenolics, ferric reducing antioxidant power and antioxidant enzyme activities of glutathione peroxidase, catalase and superoxide dismutase values in the present study were consistent with those reported in the *Longissimus thoracis et lumborum* muscle [26], liver and plasma [27] of lambs grazing ryegrass. However, in our present study, the observation that none of the dietary phenolic compounds and antioxidant enzyme activities detected in the *Longissimus dorsi* muscle were affected by the lamb gender (Table 3) or inbreeding coefficient (Table 4) suggests that gender and linebreeding had no direct impact on the antioxidant status and deposition mechanism in the muscle tissue of MARGRA lambs. This implies that TAW sheep grazing ryegrass rich in phenolics contributed to an improved overall meat oxidative stability with similar deposition and bioavailability in ewe and ram lambs irrespective of inbreeding coefficient.

### 4.1. FMP

The FMP range of 28–39 °C and an overall mean of 34.6 ± 2.3 °C obtained for TAW in the present study is well below the range of 40.6–48.0 °C and 41.5–44.0 °C reported by Flakemore et al. [13] and Holman et al. [43] in purebred and crossbred Merino, Dorset, Black and White Suffolk sheep. The presence of double or triple bonds in the FA structure leads to lower melting points because the higher the proportions of MUFA and PUFA, the more easily such bonds can be broken and the lower the fat melting points compared to the more stable SFA with high FMP. Smith et al. [66] reported that these SFA contributed to an elevation in the hardness of fat in beef, while Flakemore et al. [13] reported that the softness or hardness of fat has safety implications for meat processors and boning room personnel in abattoirs. From our results in the present study (Table 3), the lesser the SFA concentration and more MUFA and PUFA implied a low FMP, indicating that TAW is not only a healthier meat product for consumers, but also a safe product to meat processors in the abattoir due to ease of processing. It was also apparent that elevated proportions of SFA, especially palmitic (C16:0) and stearic (C18:0) acids in ram lambs, could have been the reason for the higher FMP than in ewe lambs. While the underpinning reasons behind the observed sex differences in FMP are not definitive from our present study, we can speculate that hormonal differences between ram and ewe lambs could have possibly had an indirect influence on FMP through IMF. This is because it has been reported that in intact bovine males, testosterone binds to receptors within the muscle and increases amino acid incorporation into protein, thus increasing muscular development, growth rate and muscle mass without simultaneous increases in IMF [67,68]. The lesser the IMF, the higher the FMP, and the higher the IMF as seen in TAW ewe lambs, the lower the FMP. This would seem to explain why, in the current study, the ewe lambs had higher IMF and lower FMP than ram lambs. Further research on the likely underlying molecular mechanisms behind FMP and IMF variation through lipogenic genes controlling fat metabolism like fatty acid binding protein-4, fatty acid synthase and stearoyl-CoA desaturase currently being investigated with TAW lambs in our laboratory, would assist in shedding more light.

### 4.2. IMF

IMF influences meat palatability and contributes to its juiciness, flavour and tenderness with direct linkage between intramuscular fat deposition and gender, age, genetics and nutrition [67,68,69]. The overall mean IMF of 4.4 ± 0.2% in the current study surpasses the suggested minimum Australian threshold of 4% for lamb palatability by Pannier et al. [70] who reported an overall average IMF of 4.23 ± 0.01% in lambs from sires selected for leanness. The IMF values in TAW pasture-fed lambs in the current study are much higher than the 1.25 ± 0.22% in lot-fed Manchega lambs reported by Gomez-Cortes et al. [71] in contrast to the expectation that lambs sacrificed after 42 days in the feedlot should have higher IMF. This would most likely be a combination of both genetic and nutritional effects with TAW having a genetic predisposition for a comparatively higher and faster rate of IMF deposition in response to ryegrass pasture feeding than other breeds such as the Manchega lambs fed concentrate rations with high fibrous components. Published reports of gender differences in IMF are not unanimous in their findings. For instance, while Pannier et al. [70] and McPhee et al. [16,72] reported significant sex differences in IMF just as we also observed in TAW lambs, Okeudo and Moss [73] did not find any differences in intramuscular lipid and fatty acid profiles of sheep comprising four sex-types. In beef cattle, Cafferky et al. [67] stated that the higher IMF values in steers than intact bulls are attributed to the diminished physiological effects of androgen, which reduces plasma lipids, increases lipolysis by adipocytes and stimulates androgen receptors to directly upregulate the lipogenic gene expression of fatty acid synthase and acetyl-CoA carboxylase [74,75] while simultaneously downregulating the lipolytic gene expression of monoglyceride lipase and adipose triglyceride lipase [55]. Hence, castration contributes to improved IMF deposition through increased lipogenesis and lipid uptake while decreasing lipolysis [76]. Given the hormonal differences between ewe and ram lambs, similar genetic, physiological and biochemical pathways may be involved, and our lab is currently exploring the sequencing and expression of fatty acid binding protein-4, fatty acid synthase and stearoyl-CoA desaturase genes in TAW to unravel and better understand the underpinning mechanisms of fat metabolism.

It was quite interesting that IC had no impact on FMP and IMF (Table 3). This is very significant from an eating quality perspective because it indicates that TAW lambs can produce consistently high-quality MARGRA meat product with low FMP and high IMF regardless of linebreeding with IC in the 0–15.6% range. To our current knowledge, the present study is the first of its kind to provide a significant insight into the impact of IC on meat eating quality in lamb as the only other reported research on inbreeding was in milking cows where Carrara et al. [77] reported significant (*P* < 0.04) impact of inbreeding on milk PUFA.

### 4.3. Omega-3 Long-Chain Polyunsaturated Fatty Acids

The ingestion of n-3 LC-PUFA confers a number of health benefits, including inhibiting cardiovascular diseases, cancer, and diabetes, obesity and neurodegenerative diseases such as amyotrophic lateral sclerosis, Parkinson’s, and Alzheimer’s [78] as well as improve visual and brain development [79]. Le et al. [80] reported that Food Standards of Australia and New Zealand (FSANZ) guidelines stipulate that for any food or meat to be termed a ‘source’ of n-3 LC PUFA, its EPA and DHA levels must be greater than 30 mg per 100 g per serve. TAW lambs had 32.4 ± 8.5 mg per 100 g of muscle, thus surpassing the 30 mg limit set by FSANZ for ‘source’ claim. The main FA in pastures is ALA, a precursor of the more potent n-3 LC-PUFA [81], especially EPA, DHA and DPA, which have important roles to play in human health. The observations that, as IC increased, there were no differences in FMP, IMF, C20:5n-3 (EPA), DHA, DPA, EPA+DHA, EPA+DHA+DPA and Σn-6/Σn-3 ratio and that increases in C18:3n-3 (ALA), MUFA, PUFA, C18:1, C18:2n-6 and C18:3n-6 were observed as IC decreased, indicate that linebreeding in the 0–15.6% range is not in any way detrimental to consistency in health-promoting n-3 LC-PUFA in TAW lambs. Such observations represent the first piece of experimental evidence regarding the impact of IC on omega-3 FA. Our data herein provide scientific evidence that TAW MARGRA lamb contains higher levels of health beneficial n-3 LC-PUFA than in other Australian lamb breeds previously reported by Ponnampalam et al. [82,83,84,85,86], Flakemore et al. [87], Knight et al. [88,89], De Brito et al. [90] and Fowler et al. [91]. Sex has been shown to influence heart and muscle FA composition, although the differences were restricted to only a few FA [45]. Previous studies have attributed FA variations due to sex as arising from sex-linked hormonal differences, which affect development and rumen biohydrogenation [45].

## 5. Conclusions

The results obtained from this study provide the first detailed scientific evidence of TAW MARGRA lamb with low SFA, high IMF, MUFA, n-3 LC-PUFA and lower FMP. Therefore, the meat from TAW lambs provides anecdotal and scientific evidence for adequate meat oxidative stability and human health benefits associated with n-3 LC-PUFA to consumers. This study clearly provides a scientific confirmation of the unique meat eating quality traits of TAW lambs. Based on nutritional value to consumers, this study reinforces the health benefits derived from consuming TAW MAGRA lamb in view of its high EPA, DHA and DPA contents. This high n-3 LC-PUFA profile of TAW MARGRA lamb has put this breed well ahead of others in terms of healthy meat products. Our findings clearly show significant gender variation between ram and ewe lambs. The lower SFA and higher MUFA and PUFA contents make MARGRA lamb fats very soft and smooth melting in the mouth without sticking to the palate due to its low FMP and healthier composition. This study provides evidence that IC is inconsequential in affecting antioxidant status, IMF, FMP and n-3 LC-PUFA in linebred and pasture-fed TAW sheep. This is because the observed variation in individual fatty acids was mainly driven by gender differences between ewes and rams, hence the need to accept the tested hypothesis. The practical implication is that health-conscious meat consumers are reassured by the scientific evidence herein of the consistency in the eating quality of MARGRA lamb brand from TAW sheep regardless of its linebred origin.

## Figures and Tables

**Table 1:** Nutrient and phenolic antioxidant compositions of ryegrass pastures grazed by Tattykeel Australian White lambs ^1^.

Nutrient	Composition (% DM)
DM	20.7
CP	19.0
ADF	26.5
NDF	30.9
EE	1.8
Ash	6.8
%TDN	62.5
DE (Mcal/kg)	2.8
ME (MJ/kg)	9.4
Phenolic Antioxidants:	
FCTP (mg GAE/g)	1.631
FRAP (mmol Fe^2+^ E/g)	6.572

^1^ DM: dry matter; NDF: neutral detergent fibre; ADF: acid detergent fibre; EE: ether extract; CP: crude protein; %TDN [40]: total digestible nutrients, calculated as (% of DM) = 82.38 − (0.7515 × ADF [% of DM]). ME [41]: metabolizable energy, calculated by converting %TDN to digestible energy (DE [Mcal/kg] = %TDN × 0.01 × 4.4) which was converted as ME = (DE (Mcal/kg) × 0.82) × 4.185; FCTP: Folin–Ciocalteu total phenolics; GAE: gallic acid equivalents; FRAP: ferric reducing antioxidant power.

**Table 2:** Fatty acid composition of grazed ryegrass pasture.

Fatty Acid	% Total Fatty Acids
14:0	0.6
15:0	0.2
16:1n-9c	0.0
16:1n-7c	0.2
16:0	15.7
17:0	0.5
18:2n-6 LA	14.8
18:3n-3 ALA	57.6
18:1n-9c	1.0
18:1n-7c	0.2
18:1n-7t	0.0
18:0	0.1
20:4n-6 ARA	0.0
20:5n-3 EPA	0.0
20:3n-6	0.1
20:4n-3	0.1
20:2n-6	0.1
20:0	1.6
22:5n-6 DPA-6	0.0
22:6n-3 DHA	0.0
22:5n-3 DPA-3	0.0
22:0	1.0
23:0	0.3
24:0	0.9
∑SFA	20.9
∑MUFA	4.9
∑PUFA	73.1
∑n-3 LC-PUFA	0.1
∑n-3 PUFA	58.0
∑n-6 PUFA	15.2
∑other FA	1.0
n-6/n-3	0.3

LA, linoleic acid; ALA, α-linolenic acid; EPA, eicosapentaenoic acid; DHA, docosahexaenoic acid; DPA, docosapentaenoic acid; ARA, arachidonic acid; ΣSFA, total saturated fatty acids; ΣMUFA, total monounsaturated fatty acids; and total polyunsaturated fatty acids (ΣPUFA). ∑SFA is the sum of 14:0, 15:0, 16:0, 17:0, 18:0, 20:0, 21:0, 22:0, 23:0, 24:0; ∑MUFA is the sum of 14:1, 16:1n-13t, 16:1n-9, 16:1n-7, 16:1n-7t, 16:1n-5c, 17:1n-8+a17:0, 18:1n-9, 18:1n-7t, 18:1n-5, 18:1n-7, 18:1a, 18:1b, 18:1c, 19:1a, 19:1b, 20:1n-11, 20:1n-9, 20:1n-7, 20:1n-5, 22:1n-9, 22:1n-11, 24:1n-9; ∑PUFA is the sum of 18:4n-3, 18:3n-6, 18:2n-6, 18:3n-3, 20:3, 20:4n-3, 20:4n-6, 20:5n-3, 20:3n-6, 20:2n-6, 22:6n-3, 22:5n-3, 22:5n-6, 22:4n-6; ∑n-3 LC-PUFA is the sum of 20:5n-3, 20:4n-3, 22:6n-3, 22:5n-3; ∑n-3 PUFA is the sum of 18:3n-3, 18:4n-3, 20:4n-3, 20:5n-3, 22:6n-3, 22:5n-3; ∑n-6 PUFA is the sum of 18:2n-6, 18:3n-6, 20:4n-6, 20:3n-6, 20:2n-6, 22:5n-6, 22:4n-6; ∑other FA is the sum of other individual FA present at <0.1% except ARA, DHA, EPA, and DPA.

**Table 3:** Effect of gender (Means ± s.d.) on fat melting point, intramuscular fat, fatty acids, antioxidant phenolics and enzyme activities in the *Longissimus dorsi* muscle of ryegrass-fed Tattykeel Australian White (TAW) lambs ^1^.

Variable	Ram (*n* = 47)	Ewe (*n* = 100)	Overall (*n* = 147)	*P*-Value
**Fat Melting Point (°C)**	35.5 ± 1.5	34.2 ± 2.4	34.6 ± 2.3	0.0001
**Intramuscular fat (%)**	3.4 ± 0.3	4.4 ± 1.4	4.1 ± 1.3	0.0001
**FCTP (mg GAE/g)**	1.142 ± 0.0036	1.171 ± 0.0042	1.156 ± 0.0039	0.4723
**FRAP (mmol Fe ^2+^ E/g)**	5.481 ± 0.0172	5.605 ± 0.0198	5.543 ± 0.0185	0.2982
**GSH-Px (U/g)**	0.085 ± 0.0012	0.091 ± 0.0024	0.088 ± 0.0018	0.0921
**Cat (U/g)**	39.8 ± 1.3	40.1 ± 1.5	40.0 ± 1.4	0.0882
**SOD (U/g)**	63.8 ± 5.7	64.9 ± 6.1	64.4 ± 5.9	0.1566
**Fatty Acids (mg /100 g)**				
**C12:0**	0.1 ± 0.5	0.0 ± 0.0	0.0 ± 0.3	0.1453
**C13:0**	3.2 ± 5.4	0.8 ± 3.2	1.5 ± 4.2	0.0009
**C14:0**	438.7 ± 492.2	153.8 ± 291.9	244.9 ± 389.7	0.0001
**C14:1**	11.9 ± 15.7	3.0 ± 5.8	5.8 ± 10.9	0.0001
**C15:0**	168.6 ± 172.2	46.1 ± 98.0	85.3 ± 138.3	0.0001
**C16:0**	3321.2 ± 2631.5	1093.0 ± 1457.6	1805.4 ± 2170.2	0.0001
**C16:1**	260.7 ± 237.0	94.6 ± 134.1	147.7 ± 189.5	0.0001
**C17:0**	321.0 ± 307.3	109.1 ± 237.7	176.8 ± 279.1	0.0001
**C17:1**	233.0 ± 247.8	75.3 ± 137.5	125.7 ± 194.0	0.0001
**C18.0**	2692.0 ± 2283.4	1016.9 ± 1649.3	1552.5 ± 2025.2	0.0001
**C18:1**	4942.6 ± 4041.0	1920.3 ± 2489.0	2886.6 ± 3368.4	0.0001
**C18:2 n-6 LA**	423.5 ± 266.6	125.4 ± 80.0	220.7 ± 214.9	0.0001
**C18:3 n-3 ALA**	262.6 ± 209.0	72.5 ± 81.6	133.3 ± 161.9	0.0001
**C18:3 n-6**	2.1 ± 4.3	2.4 ± 6.5	2.3 ± 5.9	0.8014
**C18:4 n-3**	3.7 ± 8.6	1.6 ± 10.1	2.3 ± 9.7	0.2262
**CLA**	117.9 ± 129.7	63.7 ± 190.0	81.1 ± 174.4	0.0788
**C19:1**	39.1 ± 41.4	14.3 ± 26.3	22.2 ± 33.8	0.0001
**C20:0**	20.3 ± 18.4	7.3 ± 10.8	11.4 ± 15.0	0.0001
**C20:1**	26.3 ± 28.4	8.1 ± 12.2	13.9 ± 20.7	0.0001
**C20:2 n-6**	7.9 ± 8.9	2.1 ± 3.1	4.0 ± 6.2	0.0001
**C20:3**	8.4 ± 4.1	11.7 ± 12.7	10.6 ± 10.8	0.0839
**C20:3 n-6**	8.8 ± 4.9	6.1 ± 2.1	7.0 ± 3.5	0.0001
**C20:4 n-3**	4.8 ± 7.3	2.1 ± 1.5	3.0 ± 4.5	0.0005
**C20:4 n-6**	36.4 ± 14.2	33.7 ± 8.0	34.6 ± 10.4	0.1473
**C20:5 n-3 (EPA)**	26.0 ± 8.5	24.3 ± 5.2	24.9 ± 6.5	0.1402
**C21:0**	2.0 ± 2.5	0.6 ± 1.4	1.0 ± 1.9	0.0001
**C22:0**	2.8 ± 3.3	2.3 ± 1.3	2.5 ± 2.2	0.1342
**C22:1**	0.4 ± 1.2	0.8 ± 1.6	0.7 ± 1.5	0.1613
**C22:4 n-6**	0.6 ± 1.2	1.4 ± 0.5	1.2 ± 0.9	0.0001
**C22:5 n-3 (DPA)**	22.5 ± 11.6	25.2 ± 8.0	24.4 ± 9.4	0.097
**C22:5 n-6**	0.0 ± 0.1	0.2 ± 0.2	0.1 ± 0.2	0.0001
**C22:6 n-3(DHA)**	5.8 ± 3.7	8.3 ± 2.7	7.5 ± 3.2	0.0001
**C23:0**	2.5 ± 2.3	2.5 ± 0.7	2.5 ± 1.4	0.7207
**C24:0**	2.2 ± 2.0	2.8 ± 0.9	2.6 ± 1.4	0.008
**C24:1 n-9c**	1.7 ± 2.1	3.9 ± 1.8	3.2 ± 2.1	0.0001
**EPA+DHA**	31.9 ± 11.3	32.6 ± 7.0	32.4 ± 8.5	0.6265
**EPA+DHA+DPA**	54.4 ± 21.8	57.9 ± 13.6	56.7 ± 16.7	0.2388
**SFA**	6971.2 ± 5684.7	2434.4 ± 3700.9	3884.9 ± 4896.6	0.0001
**MUFA**	2120.3 ± 2772.9	5515.7 ± 4577.1	3205.9 ± 3786.7	0.0001
**PUFA**	380.7 ± 331.9	931.1 ± 614.7	556.7 ± 510.0	0.0001
**PUFA/SFA**	0.2 ± 0.1	0.2 ± 0.1	0.2 ± 0.1	0.0036
**∑n-3 PUFA**	134.1 ± 96.5	325.5 ± 231.7	195.3 ± 176.7	0.0001
**∑n-6 PUFA**	479.3 ± 279.5	171.3 ± 85.2	269.8 ± 224.3	0.0001
**n-6/ n-3 PUFA**	1.6 ± 0.5	1.4 ± 0.3	1.5 ± 0.4	0.0001

^1^ FCTP: Folin–Ciocalteu Total Phenolics; GAE: Gallic acid equivalents; FRAP: Ferric reducing antioxidant power. Antioxidant enzyme activities of GSH-Px: glutathione peroxidase, Cat: catalase (Cat) and SOD: superoxide dismutase. ∑SFA sum of saturated FAs: C12:0+C13:0+C14:0CC14:0+C15:0+C15:0+C15:0+C16:0+C17:0+C18:0+C20:0+C21:0+C22:0+C23:0+C24:0; ∑MUFA sum of monounsaturated FAs: C14:1+C16:1+C17:1+C18:1+C19:1+C20:1+C21:1+C22:1+C24:1. ∑PUFA is the sum of polyunsaturated FA: C18:2 n-6+ C18:3 n-3+ C18:3 n-6+ C18:4 n-3+CLA+C20:2 n-6+C20:3+C20:3 n-6+C20:4 n-3+C22:4 n-6+C20:5 n-3+C22:5 n-3+C22:5 n-6+C22:6 n-3. ∑n-6 PUFA is the sum of n-6 PUFA: C18:2 n-6+C18:3 n-6 +C20:2 n-6+C20:4 n-6+C20:3 n-6+C20:4 n-6+C22:5 n-6. ∑n-3 PUFA is the sum of n-3 PUFA: C18:3 n-3+C18:4 n-3+C20:4 n-3+C20:5 n-3+C22:5 n-3+C22:6 n-3.

**Table 4:** Effect of inbreeding coefficients (Means ± s.d.) on fat melting point, intramuscular fat, fatty acids, antioxidant phenolics and enzyme activities in the *Longissimus dorsi* muscle of ryegrass-fed TAW lambs ^1^.

	Inbreeding Coefficient (%)			
Variable	Low (0–5) (*n* = 49)	Medium(6–10) (*n* = 49)	High (Above 10) (*n* = 49)	*P*-Value
**Fat Melting Points (°C)**	34.9 ± 2.1	34.2 ± 2.4	34.8 ± 2.8	0.225
**Intramuscular fat**	4.1 ± 1.4	4.0 ± 1.2	4.1 ± 1.0	0.9148
**FCTP (mg GAE/g)**	1.271 ± 0.0014	1.269 ± 0.0019	1.290 ± 0.0014	0.8524
**FRAP (mmol Fe ^2+^ E/g)**	6.018 ± 0.0027	6.083 ± 0.0045	6.102 ± 0.0086	0.2352
**GSH-Px (U/g)**	0.091 ± 0.0041	0.086 ± 0.0036	0.089 ± 0.0062	0.0896
**Cat (U/g)**	40.5 ± 1.8	40.1 ± 1.6	40.7 ± 1.9	0.1843
**SOD (U/g)**	64.8 ± 5.7	65.0 ± 5.9	64.5 ± 5.3	0.0972
**Fatty Acids (mg/100 g)**				
**C12:0**	0.0 ± 0.4	0.0 ± 0.0	0.0 ± 0.0	0.6757
**C13:0**	2.3 ± 4.6	0.7 ± 3.5	0.0 ± 0.0	0.0574
**C14:0**	340.3 ± 479.9	127.5 ± 176.7	94.6 ± 50.8	0.0033
**C14:1**	7.7 ± 11.9	3.6 ± 9.4	2.4 ± 1.7	0.0609
**C15:0**	116.6 ± 152.0	47.7 ± 112.7	27.1 ± 22.9	0.0074
**C16:0**	2377.1 ± 2509.5	1100.6 ± 1410.2	923.7 ± 527.9	0.0013
**C16:1**	194.1 ± 215.9	90.9 ± 135.4	71.8 ± 38.3	0.0033
**C17:0**	241.4 ± 322.4	98.9 ± 193.4	61.0 ± 32.9	0.006
**C17:1**	165.6 ± 202.9	78.3 ± 178.8	46.6 ± 26.2	0.0174
**C18.0**	2130.6 ± 2447.3	840.2 ± 938.8	655.2 ± 288.8	0.0004
**C18:1**	3704.7 ± 3831.0	1885.3 ± 2423.3	1552.9 ± 690.9	0.0036
**C18:2 n-6 (LA)**	271.1 ± 244.2	158.9 ± 156.5	139.0 ± 65.3	0.0053
**C18:3 n-3 (ALA)**	170.3 ± 179.0	89.0 ± 129.2	63.7 ± 33.0	0.0067
**C18:3 n-6**	3.1 ± 7.8	1.3 ± 0.9	1.5 ± 0.8	0.032
**C18:4 n-3**	3.1 ± 11.5	1.4 ± 7.0	0.4 ± 0.3	0.5311
**CLA**	114.4 ± 219.4	40.4 ± 75.8	24.9 ± 13.7	0.032
**C19:1**	29.4 ± 37.4	13.6 ± 27.5	9.1 ± 5.2	0.0139
**C20:0**	15.5 ± 17.7	6.5 ± 8.4	5.2 ± 2.7	0.001
**C20:1**	18.4 ± 22.3	8.7 ± 17.9	5.2 ± 2.2	0.0124
**C20:2 n-6**	5.3 ± 7.2	2.2 ± 4.3	2.0 ± 2.1	0.0092
**C20:3**	11.0 ± 14.3	10.2 ± 2.7	9.4 ± 2.7	0.8782
**C20:3 n-6**	7.6 ± 3.9	6.2 ± 2.7	5.6 ± 1.1	0.8782
**C20:4 n-3**	3.6 ± 5.3	2.3 ± 3.1	1.6 ± 0.6	0.171
**C20:4 n-6**	32.8 ± 11.6	36.6 ± 8.4	38.0 ± 8.3	0.0737
**C20:5 n-3 (EPA)**	24.3 ± 7.0	25.8 ± 5.9	23.0 ± 4.8	0.3091
**C21:0**	1.6 ± 2.3	0.4 ± 0.9	0.1 ± 0.2	0.0009
**C22:0**	3.0 ± 2.7	1.8 ± 1.1	1.6 ± 0.8	0.0055
**C22:1**	0.8 ± 1.9	0.6 ± 0.9	0.3 ± 0.3	0.5598
**C22:4 n-6**	1.2 ± 1.0	1.2 ± 0.7	1.3 ± 0.7	0.9005
**C22:5 n-3 (DPA)**	24.7 ± 11.5	23.9 ± 5.7	23.3 ± 5.0	0.8515
**C22:5 n-6**	0.1 ± 0.2	0.2 ± 0.2	0.2 ± 0.3	0.3705
**C22:6 n-3(DHA)**	7.4 ± 3.8	7.6 ± 2.4	7.5 ± 3.1	0.9816
**C23:0**	2.7 ± 1.7	2.7 ± 1.0	2.0 ± 1.1	0.2737
**C24:0**	2.7 ± 1.5	2.5 ± 1.2	2.3 ± 1.3	0.674
**C24:1 n-9c**	2.9 ± 2.1	3.5 ± 2.1	3.7 ± 2.1	0.2498
**EPA+DHA**	31.8 ± 9.5	33.4 ± 7.1	30.6 ± 7.6	0.4708
**EPA+DHA+DPA**	56.5 ± 19.8	57.3 ± 11.9	53.8 ± 12.4	0.8738
**SFA**	5231.3 ± 5786.8	2228.5 ± 2775.3	1772.7 ± 913.9	0.0007
**MUFA**	4123.6 ± 4281.0	2084.4 ± 2782.6	1691.9 ± 761.3	0.0036
**PUFA**	680.1 ± 577.2	407.1 ± 373.2	341.5 ± 105.3	0.0037
**PUFA/SFA**	0.2 ± 0.1	0.2 ± 0.1	0.2 ± 0.0	0.0781
**∑n-3 PUFA**	233.4 ± 196.1	149.9 ± 141.9	119.6 ± 27.5	0.0114
**∑n-6 PUFA**	321.2 ± 255.7	206.6 ± 162.3	187.6 ± 71.6	0.0067
**n-6/ n-3 PUFA**	1.5 ± 0.4	1.5 ± 0.3	1.5 ± 0.3	0.8487

^1^ Abbreviations are the same as in Table 3.
